# Use of Electroneuromyography in the Diagnosis of Neurogenic Thoracic Outlet Syndrome: A Systematic Review and Meta-Analysis

**DOI:** 10.3390/jcm11175206

**Published:** 2022-09-02

**Authors:** Pauline Daley, Germain Pomares, Raphael Gross, Pierre Menu, Marc Dauty, Alban Fouasson-Chailloux

**Affiliations:** 1 Service de Médecine Physique et Réadaptation Locomotrice et Respiratoire, CHU Nantes, Nantes Université, 44093 Nantes, France; 2 Service de Médecine du Sport, CHU Nantes, Nantes Université, 44093 Nantes, France; 3 Institut Européen de la Main, Hôpital Kirchberg, 2540 Luxembourg, Luxembourg; 4 Laboratoire Motricité-Interactions-Performance, CHU Nantes, Nantes Université, MIP, EA4334, 44000 Nantes, France; 5 Institut Régional de Médecine du Sport, IRMS, 44093 Nantes, France; 6 Inserm, UMR 1229, RMeS, Regenerative Medicine and Skeleton, Nantes Université, ONIRIS, 44042 Nantes, France

**Keywords:** thoracic outlet syndrome, neurogenic, electromyography, nerve conduction

## Abstract

Neurogenic thoracic outlet syndrome (NTOS) is a disabling condition. Its diagnosis remains challenging and is mainly guided by examination. Yet, electrophysiological evaluations are the gold standard for diagnosis of entrapment syndromes. We aimed to assess the interest of electrophysiological evaluation to diagnose NTOS. A systematic literature research was performed using PubMed, ScienceDirect, Embase, Cochrane and Google Scholar databases to collect studies reporting results of electrophysiological assessment of patients with NTOS. Then, a meta-analysis was conducted. Nine studies were eligible and concerned two hundred and thirteen patients. Results were heterogenous among studies and the quality of evidence was very low to moderate. Data could not evaluate sensitivity or specificity of electrophysiological evaluations for NTOS. The meta-analysis found significantly decreased amplitudes of medial antebrachial cutaneous nerve SNAP (sensory nerve action potential), ulnar SNAP, median CMAP (compound motor action potential) and ulnar CMAP. Needle examination found abnormalities for the abductor pollicis brevis, first dorsal interosseous and adductor digiti minimi. Unlike most upper-limb entrapment syndromes, nerve conduction assessment only provided clues in favour of NTOS. Decreased amplitude for ulnar SNAP, medial antebrachial cutaneous SNAP, median CMAP and ulnar CMAP should be assessed, as well as needle examination. Larger studies are needed to evaluate the sensitivity and specificity of electrophysiology in NTOS diagnosis.

## 1. Introduction

Thoracic outlet syndrome (TOS) is a heterogenous entity including all the manifestations of intermittent or permanent compression of neurovascular structures along their pathway through the cervico-thoracic outlet [1,2,3]. This anatomical area is divided into at least three potential compression sites [4]: the interscalene triangle (compression site of the brachial plexus and the sub-clavian artery), delimited by the anterior and middle scalene muscle and the first rib; the costo-clavicular space (compression of the brachial plexus and the sub-clavian vessels), delimited posteriorly and inferiorly by the first rib, and anteriorly by the sub-clavian muscle and the inferior aspect of the clavicle; and the sub-coracoid space (compression of the axillary vessels and brachial plexus), delimited anteriorly by the tendon of pectoralis minor inserting onto the coracoid process. Intermittent and position-dependent compression of the neurovascular bundle occurs mainly in the costo-clavicular and sub-coracoid space. It can also be permanent, mainly if a NTOS is linked with a congenital variation, narrowing the interscalene triangle. Neurogenic TOS (NTOS) represents approximately 90% of patients with thoracic outlet syndrome [1,3,4]. It is an impairing and painful condition, responsible for a lack of force and endurance, both at the proximal and distal upper-limb level [5,6,7]. NTOS diagnosis remains challenging because it is based on the exclusion of other diagnoses, and on symptoms and clinical examination [2,8,9,10]. Usually, patients describe subjective symptoms such as pain (which can be cervical, located on the scapular region or in the arm or hand), paraesthesia and loss of strength. It is important to note that most patients do not actually have a clinical deficit, but only subjective symptoms. This is particularly relevant due to the sensitivity of electrophysiology. Medical imaging can sometimes help in making the diagnosis if it shows anatomical risk factors, such as a cervical rib [2,9]. Yet, the interest of imaging remains, mainly to rule out other diagnoses [8,9,10]. For example, doppler examination has proved to be of poor contribution for the diagnosis of NTOS [11,12], with a recent study showing a sensitivity of 54.5% and specificity of 67% [11]. Furthermore, results of electrodiagnosis studies have been conflicting. Indeed, some studies described the association of a very low sensory response of the medial antebrachial cutaneous (MABC) nerve (mostly T1-innervated), a low sensory nerve action potential (SNAP) amplitude over the ulnar nerve (mostly C8-innervated), and a low compound motor action potential (CMAP) amplitude of the median nerve recorded on the abductor pollicis brevis (APB) muscle (mostly C8-innervated) [13,14,15,16], as pathognomonic of NTOS. On the contrary, some other studies described these as normal electrophysiological findings [17,18]. Thus, in a recent recommendation for the diagnosis of thoracic outlet syndrome, electrophysiological assessment could be helpful, though not essential [8]. Yet, the authors advised to include the medial antebrachial nerve conduction evaluation in the case of electromyographic exam. Because of these conflicting findings about electrodiagnosis, we aimed to perform a systematic review to assess the role of electrodiagnosis in the diagnosis of NTOS.

## 2. Materials and Methods

### 2.1. Literature Search

This systematic review was registered with PROSPERO under the registration number CRD42022322405, and we used PRISMA guidelines [19,20]. We searched articles in the most commonly used medical databases: PubMed, ScienceDirect, Embase, Cochrane and Google Scholar, in March 2022. Article research extended from January 2000 to February 2022. Only studies in English language including 3 or more cases were selected. Multiple searches were carried out using the following MeSH: “thoracic outlet syndrome” AND (“electromyography” OR “nerve conduction study”). The search was performed independently by 2 assessors (P. D., A. F.-C.) to assess titles and abstracts of potentially relevant articles, and the full-text articles were retrieved. In case of doubt, a third assessor’s advice was asked (M. D.). All relevant articles were read independently in full by the two researchers (P. D., A. F.-C.) to assess whether the articles met the inclusion criteria. After the identification of key articles, their references and citation lists were also hand searched for further information sources. Reviews and meta-analyses were also analysed, to broaden the search for studies that were possibly missed through the electronic search.

### 2.2. Eligibility Criteria and Data Extraction

The inclusion criteria were: studies including 3 cases or more of thoracic outlet syndrome evaluated with standard and reproducible electrophysiological techniques. All types of electrophysiological evaluation were included. The criteria of exclusion were: patients suffering from other plexopathies or exclusive vascular TOS, and publications in languages other than English.

All the included studies were analysed, and data were collected and summarised in tables using Microsoft Excel (version 2013, Microsoft corporation, Redmond, WA, USA) including: study design, year of publication, number of subjects, type of evaluation and findings.

### 2.3. Quality Analysis

The included studies were critically appraised using the GRADE approach, so as to assess the quality of evidence of these studies [21]. This approach classified the quality of evidence into one of four levels: very low, low, moderate and high. Evidence based on observational studies began as ‘low quality’ evidence, but the strength of our confidence in the evidence could have increased due to fewer biases such as study limitations, inconsistency of results, indirectness of evidence, imprecision and reporting bias.

### 2.4. Meta-Analysis

We performed a meta-analysis for the available values of electroneuromyography results. Heterogeneity across the combined data was assessed with the use of the I² test; a value of I² > 50% was considered to indicate significant heterogeneity [22]. We also reported the among-study variance (tau²) [22]. We used the random-effect method for this meta-analysis, assuming that the evaluation of patients varied within studies with different evaluation protocols. Pooled summary statistics were calculated with a random-effect model, using the Meta-Mar online tool [23] for statistical calculations based upon mean values and standard deviations.

## 3. Results

### 3.1. Study Selection

**Figure 1:**
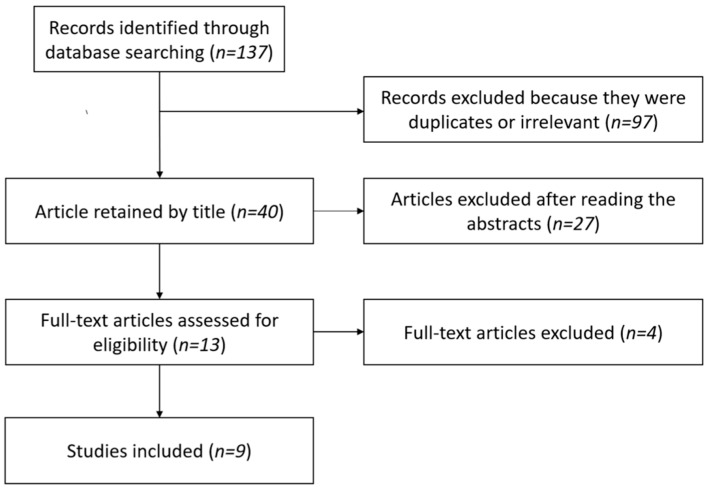
Selection of the studies according to PRISMA guidelines.

Our research found 137 results. Out of these records, we retained 40 articles by title. After removing duplicates and reading abstracts, 13 articles were assessed for full-text reading. We excluded one of them because it did not explore patients with routine electrophysiological assessment [24], and another because it included only two patients [25]. Another article was excluded because it did not differentiate NTOS from other plexopathies [26]. One article was excluded because it presented results of pre-surgery electrophysiological evaluations [27]. Finally, we included nine articles [14,17,28,29,30,31,32,33,34] (Figure 1). Three studies were eligible for meta-analysis [28,29,32]. Because they were all retrospective studies of similar methodological quality, they were not weighted by results based on methodology. Given the small number of studies included in this meta-analysis, it was not surprising to find large 95% confidence intervals.

### 3.2. Demographic Data

Studies included a total of 203 patients and 83.7% were women. Age was specified in all studies except for one [33]. Six studies provided mean ages, from 31.3 to 43.1 years old [30,32], and two provided median ages of 30.6 [31] and 29.5 [17], respectively. Demographics of the included studies are reported in Table 1.

Gillard et al. [34] studied the contribution of the somatosensory evoked potential and electromyogram results, without precision, of 31 patients with thoracic outlet syndrome. Rousseff et al. [17] discussed the utility of the somatosensory evoked potential, electroneurography and electromyography in their surgical series of TOS. These two articles did not make clear the numerical results of the nerve conduction assessments, because only the conclusions were provided. Seror et al. [32] and Machanic et al. [33] were the first to explicitly explore medial antebrachial cutaneous (MABC) nerve conduction in thoracic outlet syndrome. Ozgönenel et al. [30] reported electrophysiological values for median and ulnar explorations in non-surgical TOS in neutral and provocative positions. Tsao et al. [14] described ulnar, median and MABC nerve conduction results through percentages of abnormal amplitudes, while Mul et al. [28] and Kim et al. [29] detailed each patient’s amplitude values on both sides. Akkus et al. [31] exposed electrodiagnostic findings (median, ulnar and MABC nerve conduction, and F-waves for median and ulnar nerves), before and 3 months after surgery (first-rib resection associated to scalenectomy, plus correction of other fibrous or osseous abnormalities if found). The evaluation of the level of evidence using the GRADE approach is reported in Table 2.

### 3.3. Neurological Examination

The duration of the symptoms was specified in four studies [14,28,29,30], and mean duration ranged from 61 to 98 months. Paresthesia in the MABC nerve area (medial side of the forearm), when described, concerned 0% to 69% of the patients [14,17,28,29,32]. Sensitive symptoms of the last two digits were reported in only three studies and concerned 31% to 69% of the patients [14,28,29]. Thenar or hypothenar atrophy was variable among studies between 0% and 100% of the included cases [14,32]. In their study, Tsao et al. [14] described 100% of the patients had amyotrophy, 97% complained of hand grip weakness and 41% of numbness, pain or paraesthesia involving the medial area of the forearm.

### 3.4. Sensory Nerve Conduction Assessment

Five studies evaluated antidromic sensory conduction of the medial antebrachial cutaneous nerve using the antidromic method [14,28,29,32,33]. They all found abnormalities. They described abnormal side-to-side amplitudes (defined by a ratio of side-to-side amplitudes superior to two, when mentioned) in 57 to 95% of the cases (Table 3). When controls were evaluated, they all had normal ratios [33]. Machanic et al., in their surgical cohort, also described significant different latency responses of the MABC nerve in pre-operative NTOS patients when compared with controls [33]. The MABC nerve latency was not reported in the other studies. Median sensory nerve action potential (SNAP) amplitude was studied in five of the studies [14,17,28,29,32]. It was described as normal for all patients in four of the studies [14,17,29,32], whereas Mul et al. described 14% of patients presenting a decreased amplitude [28]. A ratio of amplitudes between asymptomatic and symptomatic sides was superior to two for 14% of the patients in the study by Mul et al. [28], and for 4% of the patients in the study by Tsao et al. [14], whereas the other three studies described normal side-to-side amplitudes for all the patients [17,29,32]. As described in Table 3, the ulnar SNAP amplitude was more often described as abnormal. Ulnar SNAP amplitude was abnormal for 0% [32] to 79% [28] of the cases when using absolute criteria, and for 0% [32] to 78% [14] when using side-to-side ratios. Rousseff et al. [17] mentioned a decrease in sensory action potential for 5% of the patients, without mentioning whether this finding was absolute or relative.

**Figure 2:**
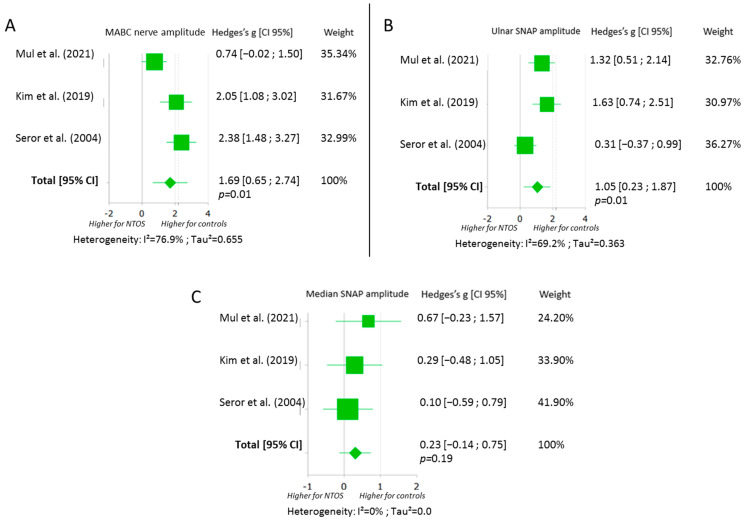
Forest plots of the sensory nerve conduction studies for MABC (**A**), ulnar (**B**) and median nerves (**C**) [28,29,32]. Abbreviations: MABC: medial antebrachial cutaneous; SNAP: sensory nerve action potential; NTOS: neurogenic thoracic outlet syndrome; CI: confidence interval.

The meta-analysis displayed in Figure 2 showed that there was a significantly higher amplitude in the sensory nerve response of MABC and ulnar nerves among controls when compared with symptomatic limbs. We calculated a Hedges’s g effect of 1.69 (0.65; 2.74) for the MABC nerve amplitude and 1.05 (0.23; 1.87) for the ulnar sensory amplitude, in favour of the control limbs. Regarding median SNAP amplitude, NTOS limbs and control limbs were statistically comparable.

### 3.5. Motor Nerve Conduction Assessment

Three studies described, from 64 to 91%, abnormal compound motor action potential (CMAP) amplitudes [14,28,29] Meanwhile, two studies described no abnormalities in electroneuromyographical evaluation regarding ulnar and median CMAP amplitudes, though they specifically explored them [17,32]. The three studies which found abnormalities in motor conduction evaluations found, from 64 to 91%, absolute abnormalities in median motor assessments, when recorded for the abductor pollicis brevis [14,28,29] (Table 3).

**Figure 3:**
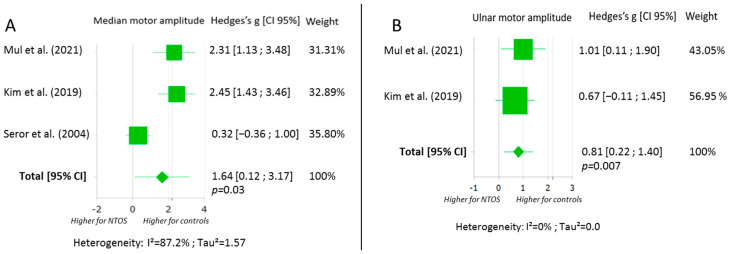
Forest plots of the motor nerve conduction studies for median (**A**) and ulnar (**B**) nerves [28,29,32]. Abbreviations: CI: confidence interval; NTOS: neurogenic thoracic outlet syndrome.

The meta-analysis displayed in Figure 3 showed that there was a significantly higher amplitude in the motor nerve response of the median and ulnar nerves among controls when compared with symptomatic limbs. We found a Hedges’s g effect of 1.64 (0.12; 3.17) for the median motor amplitude recorded on the abductor pollicis brevis, and of 0.81 (0.22; 1.40) for the ulnar motor amplitude recorded on the abductor digiti minimi, in favour of control limbs.

### 3.6. Late Responses

Gillard et al. [34] described consistently normal somatosensory evoked potentials. Rousseff et al. [17] described a delay of the N20 (negative peak at 20 ms) of the ulnar somatosensory evoked potential for one patient out of twenty. Ozgönenel et al. [30], in their series, found a statistical difference in F-wave values for median and ulnar nerves, both in neutral and provocative positions between patients and controls. However, these values were all within reference range, and the differences were all inferior to 2 ms, therefore, not clinically significant. Akkus et al. [31] found a slight but significant prolonged median F-wave difference between sides, with an unaffected side F-wave measured at 22.94 ± 1.79 ms vs. 23.98 ± 2.05 ms on the affected side (*p =* 0.015). Ulnar F-wave did not significantly differ between sides.

### 3.7. Needle Examination

Myography found abnormal results in 0% [17] to 100% [14] of the patients. It highlighted differences in recruitment between studies. Electromyographical results are reported in Table 4. Abnormal electromyographical results were counted as the number of abnormalities among all myographies, even if each muscle described was not explored in all patients. Abnormalities were predominant for the abductor pollicis brevis, which was found to be abnormal in 25% [32], 50% [14,28] and 85% [29] of the cases according to the series. The first dorsal interosseous (FDI), abductor digiti minimi (ADM), extensor indicis proprius and opponent pollicis showed frequently less denervation.

### 3.8. Sensitivity to Change

Seror et al. [32] described one patient who received pre/post-operative evaluation, with MABC nerve amplitude evolving from 32 µV to 13 µV after surgery. Variation in MABC SNAP after surgery was the primary outcome of the work of Akkus et al. [31]. A significant increase was observed post-operatively in latencies, with a conduction speed increasing from 55.1 ± 6.36 m/s to 62.15 ± 3.08 m/s after three post-operative months. All these values were within normal ranges. The ulnar sensory nerve action potential in µV and median motor amplitude in mV, were also significantly increased post-operatively (from 51.35 ± 8.95 to 58.66 ± 6.8 (*p* = 0.003) and from 12.43 ± 2.32 to 15.2 ± 2.82 (*p* = 0.0001), respectively). Machanic et al. [33] performed post-operative electrophysiological assessment of the MABC nerve in 10 patients. In seven patients out of eight who improved with surgery, the amplitude increased, and remained unchanged in the eighth one. In the two patients without clinical improvement, the amplitude decreased in one and remained unchanged in the other one. These data were not statistically significant. Ozgönenel et al. [30] found no significant modifications in F-wave latencies during provocative manoeuvres.

### 3.9. NTOS Patients with No Clinical Motor Deficit

For twenty patients, it was clearly described that they presented no motor abnormalities on neurological examination (sixteen patients in Seror et al. [32], three patients in Kim et al. [29] and one patient in Mul et al. [28]). For these patients, the mean ratio ± standard deviation (SD) for affected/unaffected limbs was 0.97 ± 0.21 for median CMAP amplitude, 0.99 ± 0.17 for median sensory nerve amplitude and 0.92 ± 0.25 for ulnar sensory nerve amplitude. The mean ratio ± SD for affected/unaffected limbs was 0.29 ± 0.23 for MABC nerve amplitude. This meant that MABC nerve amplitude was significantly decreased in those patients with no motor symptoms. One patient in Kim et al. [29] and four patients in Seror et al. [32] had neuropathic recruitment alteration on the abductor pollicis brevis, without denervation potentials. This represented 20% of the abnormality for myographies among patients with no motor deficit.

## 4. Discussion

In this review and meta-analysis, in case of NTOS, we found modifications concerning the potentials of action amplitudes of the MABC nerve, the ulnar nerve sensory conduction, and the ulnar and the median CMAP amplitudes. These decreases in amplitude seem consistent due to anatomical facts, showing signs of lower plexopathy that could be induced by intermittent compression. Indeed, the MABC nerve mainly derives from the T1 root. Several recent studies corroborated this MABC conduction decrease, which might be the only electrophysiological abnormality supporting the diagnosis of NTOS [13,32,33,35]. In the case of other abnormalities, studies have also supported axonal lower plexopathy with C8–T1 damage: abnormalities in ulnar SNAP and in median CMAP seem more frequent than the CMAP of the ulnar nerve; abnormal myographies for the APB (mainly T1-innervated) and FDI (C8-T1) were also described [13,35]. Among the six studies that included reporting the percentage of normal electrophysiology findings, three of them described 0% normality [14,28,32], while one described 2% [33], another 8% [29] and a last one counted 90% normality [17]. However, most of the studies presented methodological limits, with a very low to moderate quality of evidence.

Machanic et al. [33] tried to develop criteria for NTOS electrodiagnosis through MABC nerve evaluation. However, they did not clearly explain the demographic characteristics of their controls. Indeed, no data regarding their age, weight and comparability with the symptomatic group were described in their study. Their results were not clearly confirmed by other studies [14,28,29,32]. In their surgical cohort, Machanic et al. [33] also described significantly different latency in the response of the MABC nerve. Yet, the neurovascular bundle intermittent compression in NTOS always occurs proximally to where the stimulation is performed; it is why it was very surprising to find these data, and an isolated conduction block of the MABC nerve in the forearm seemed difficult to justify. In the case of association with a decrease in amplitude, it can be a secondary sign of an axonal lesion. The other studies only reported MABC nerve amplitude, so we could not know if its latency was modified [14,28,29,32]. The heterogeneity of the evaluation criteria, and the findings, did not enable us to establish a mean proportion of the overall sensitivity and specificity of electroneuromyography in NTOS. This heterogeneity of results could be explained by several factors. Firstly, the populations were not all comparable. This was due to the differences in diagnostic criteria of TOS. Indeed, the diagnosis of NTOS was completed with variations among studies. So, the results had to be interpreted critically and cautiously. NTOS severity varied as well. For example, the study by Rousseff et al. [17] only considered “surgically verified TOS”, which is currently not an admitted criterion for the diagnosis of NTOS [4,8,9,36,37]. In some studies, inclusion criteria overlapped with the evaluation. Indeed, in the study by Kim et al. [29], one of the inclusion criterion was “paresthesia in the medial side of forearm with or without digit four and five, or obvious hypesthesia in the innervated area of the MABC nerve or ulnar nerve”. In these patients, it seemed not surprising that exploration of the SNAP of the MABC nerve found a decrease in amplitude in 11 out of 12 patients (92%). Surgical series might have included patients with more severe symptoms, which could have increased heterogeneity among studies. Recruitment also differed depending on the cohort (surgical vs. non-surgical). These things considered, the cohorts with a large number of clinical abnormalities, such as wasted C8–T1-innervated muscles [14] or hypoesthesia of the last two digits and the medial side of the forearm [28], might have shown more electrophysiological abnormalities. The description of “Gilliatt-Sumner-hand” in 1970, associating a cervical rib with hypothenar or thenar atrophy, is currently considered one of the many features of thoracic outlet syndrome [8,15,27]. This finding is scarce in wide cohorts of NTOS patients, even though weakness and decreased endurance have been described [5,6]. Most patients display only subjective symptoms linked to intermittent compression, and have no clinical deficit on examination [8,9]. Secondly, electrodiagnostic results differ and can be dependent on the practitioner. It is especially the case for the MABC nerve, which is a small sensory nerve. Its assessment can be technically challenging, due to low amplitude responses and, muscle and nerve artefacts. Its exploration in NTOS was firstly described by Seror et al. [32] in 2004, following the work of Le Forestier et al. [38] in 2000. Its optimal exploration remains controversial, with studies showing that the best stimulation site for its exploration should be either on the antecubital fossa [33] or more proximal [39,40]. Its optimal exploration seems to be antidromic [39,40,41]. Myography can also be arduous to interpret. In fact, pain can modify the motor units discharge rate [42,43,44]. Moreover, abnormalities regarding needle myography can be over-estimated because needle examination is often guided by clinical suspicion, and we may suspect that not all patients have an exhaustive needle electromyography. Abnormalities in needle examinations are predominantly found in muscles that are frequently explored in routine myographies (APB, FDI, ADM). Differences can also be due to considerable variations in brachial plexus anatomy, as described previously [45,46,47]. Moreover, muscles are often innervated by two spinal segments, and electrodiagnostic evaluations cannot provide information on segmental variation [47,48,49]. Other abnormalities might have been mis-estimated because they were not looked for. This may be partly explained by the fact that in retrospective studies, the protocols for nerve conduction evaluation and myography were not uniform [14,17,28,29,32]. In practice, the physician needs to make choices regarding which nerves and muscles to examine, and time limits the number of muscles that can be sampled. Therefore, the physician may choose muscles that seem weak on clinical evaluation and this might lead them to mis-estimate the percentage of abnormalities. For example, the study by Kim et al. [29] was the only one to describe needle examination for the extensor indicis proprius, and found nine abnormal explorations out of thirteen explorations of this nerve. No other study described the evaluation of this muscle, which made it difficult to evaluate the frequency of this finding. The physiopathology of NTOS itself can also explain these variations in sensitivity. Unlike most upper-limb entrapment syndromes, it is important to keep in mind that NTOS symptoms are usually linked to intermittent irritation of the brachial plexus, without permanent compression. Finally, conclusions can differ due to the use of different cut-offs in normality. It can be challenging if a study does not clearly explain its numerical values to exploit its results, as in the studies by Rousseff et al. [17] and Gillard et al. [34]. Because of these elements, this study could not determine the sensitivity of electrophysiology in diagnosing neurogenic thoracic outlet syndrome. We could only enhance the high variation in its sensitivity among studies. The results of electrophysiological studies on patients with no motor abnormalities are noteworthy. We found that for patients with no motor deficits on clinical examination, MABC nerve amplitude was significantly lowered, and myography was altered in 20% of the cases. This means electrophysiology, especially MABC nerve evaluation, could be of interest for patients with only subjective or sensitive symptoms, to reveal infraclinical axon loss. This sub-group of patients with NTOS can represent most patients with NTOS when making the diagnosis. Their optimal clinic and paraclinic evaluations are crucial because they could allow to better diagnose milder forms of NTOS. Motor deficit is usually evaluated on examination with the MRC scale, but a more precise evaluation could be performed in this indication. Some recent data indicated that a systematic evaluation of strength with a grip and pinch gauge could reveal subclinical strength loss for patients with NTOS [50].

Due to the low number of included studies, their heterogeneity, lack of power and methodological quality, and the high inconsistency of the results, the conclusions of this meta-analysis should be taken with extreme caution. It seems hazardous to consider electrophysiology as a true tool for NTOS diagnosis. In our experience, electrophysiology is proposed when a double-crush syndrome is suspected, or when we need to rule out another diagnosis. Indeed, the normality of the electrophysiological evaluation does not rule out NTOS. We could wonder if standard electromyography is sufficient in this pathology, or if dynamic electrophysiological evaluation could be of use, as NTOS is mainly linked with intermittent irritation. Ozgönenel et al. [30] performed electromyography in the neutral and provocative position; however, their evaluation was only based upon F-wave.

Some recent studies tried to develop imaging contribution in NTOS. Standard MRI imaging can reveal anatomic variations; however, in a recent study with surgically treated patients, there was a low correlation between MRI data and pre-operative findings [51]. Sensitivity and specificity were, respectively, 41% and 33%. Magnetic resonance neurography seems to have an interest for the positive diagnosis of NTOS and to highlight a zone of compression or irritation of the brachial plexus [52,53], but is not frequently used in clinical practice.

Given the findings of this systematic review and meta-analysis, we estimate that more data are necessary to determine more precisely the place of electroneuromyography in NTOS diagnosis.

## 5. Conclusions

This review and meta-analysis found asymmetrical amplitudes of SNAP for MABC and ulnar nerves, as well as asymmetrical amplitudes of CMAP for ulnar and median nerves in patients with NTOS. There was a relative paucity of high-quality studies in this field, and the evidence supporting these results was insufficient. We cannot evaluate precisely, the sensitivity of electrophysiological assessment in thoracic outlet syndrome. Abnormalities in needle examinations regarded mainly the APB, FDI and ADM. Late responses such as F-waves and somatosensory evoked potentials did not show their interest in this context. Due to the heterogeneity of the diagnosis of NTOS among studies, and methodological biases, important differences in electrophysiological findings were described. This review highlighted an important variability in the sensitivity of electrophysiology in NTOS. Electroneuromyography cannot be considered a true diagnosis tool for NTOS at this point, though it remains useful to rule out differential diagnosis in this context. It could be interesting for patients with no clinical motor deficit, as we highlighted abnormalities in amplitudes of SNAP for MABC nerves and showed myography abnormalities in this population. Larger studies with standardised electroneuromyographic protocols and bi-lateral evaluations are needed to evaluate the sensitivity and specificity of electrophysiology in NTOS, and its contribution to confirm objectively the diagnosis. Data suggested that there might have been an interest of electrophysiological evaluations in the patients’ follow-up, but these data need further confirmation.

## Figures and Tables

**Table 1:** Demographic data of the included studies.

Studies	Population	Symptomatic Limbs (*n*)	Mean Age (Years)	Gender (M/F)	Duration of Symptoms (Months)	Diagnostic Criteria
Mul et al. (2021) [28]	Not specified	14	36.4	2/12	127	Clinical + NCS
Kim et al. (2019) [29]	Surgical and non-surgical	13	40.3	3/10	70.6	Clinical
Akkus et al. (2018) [31]	Surgical	15	30.6 *	3/12	NA	Clinical
Tsao et al. (2014) [14]	Surgical	32	40.7	2/30	61	NCS
Ozgönenel et al. (2012) [30]	Non-surgical	21	31.3	3/18	62	Clinical
Machanic et al. (2008) [33]	Surgical	41	NA	9/32	NA	Clinical
Seror et al. (2004) [32]	Non-surgical	16	43.1	2/14	NA	NCS
Rousseff et al. (2004) [17]	Surgical	20	29.5 *	4/16	NA	Surgical TOS
Gillard et al. (2001) [34]	Non-surgical	31	37.1	5/26	NA	Clinical + NCS + imaging

Abbreviations: NCSs: nerve conduction studies; NA: not available; TOS: thoracic outlet syndrome; *: median.

**Table 2:** Evaluation of the level of evidence using the GRADE approach.

Studies	Design	Limitations in Study Design or Execution	Inconsistency of Results	Indirectness of Evidence	Imprecision	Quality of Evidence
Mul et al. (2021) [28]	Retrospective case series	+	-	-	-	Low
Kim et al. (2019) [29]	Retrospective case series	+	-	-	-	Low
Akkus et al. (2018) [31]	Prospective case series	+	+	-	+	Very low
Tsao et al. (2014) [14]	Retrospective case series	+	-	-	+	Low
Ozgönenel et al. (2012) [30]	Prospective case series	-	-	-	-	Moderate
Machanic et al. (2008) [33]	Prospective case series	-	-	-	+	Low
Seror et al. (2004) [32]	Retrospective case series	-	-	-	-	Low
Rousseff et al. (2004) [17]	Retrospective case series	++	-	-	++	Low
Gillard et al. (2001) [34]	Prospective case series	+	-	+	++	Very low

Abbreviations: ++: important bias, +: bias, -: no bias.

**Table 3:** Sensory and motor nerve conduction studies.

Studies	Number of Symptomatic Limbs	Abnormal MABC Nerve Amplitude (Side-to-Side Ratio for Abnormality)	Absolute Abnormal SNAP Ulnar Amplitude (Chosen Cut-Off)	Relative Abnormal SNAP Ulnar Amplitude (Side-to-Side Ratio for Abnormality)	Abnormal CMAP Median Amplitude (APB), Absolute (Chosen Cut-Off)	Abnormal CMAP Median Amplitude (APB), Relative (Side-to-Side Ratio for Abnormality)	Abnormal CMAP Ulnar Amplitude, Absolute (Chosen Cut-Off)	Abnormal CMAP Ulnar Amplitude, Relative (Side-to-Side Ratio for Abnormality)
Mul et al. (2021) [28]	14	50% (2)	79% (19.3 µV)	71% (2)	64% (6.2 µV)	100% (2) *	28% (8.4 µV)	20% (2) **
Kim et al. (2019) [29]	13	92% (2)	46% (age-stratified norms)	58% (2)	85% (age-stratified norms)	75% (2)	54% (age-stratified norms)	8% (2)
Tsao et al. (2014) [14]	32	95% (2)	6% (age-stratified norms)	78% (2)	91% (age-stratified norms)	97% (2)	3.1% (age-stratified norms)	38% (2)
Machanic et al. (2008) [33]	41	61% (2)	NA	NA	NA	NA	NA	NA
Seror et al. (2004) [32]	16	94% (2)	NA	NA	0% (not mentioned)	0% (2)	0% (not mentioned)	0% (not mentioned)
Rousseff et al. (2004) [17]	20	NA	5% (criteria not mentioned)		0% (not mentioned)	0% (not mentioned)	0% (not mentioned)	0% (not mentioned)

Abbreviations: MABC: medial antebrachial cutaneous; SNAP: sensory nerve action potential; NA: not available; APB: abductor pollicis brevis; CMAP: compound motor action potential; **: *n* = 6; *: *n* = 5.

**Table 4:** Percentage of abnormal needle electromyographies per muscle.

Studies	Myographies (*n*)	APB (Number of Evaluations)	FDI (Number of Evaluations)	ADM (Number of Evaluations)	Other (Number of Evaluations)
Mul et al. (2021) [28]	14	50% (9)	86% (13)	36% (7)	ED 29% (8), FCR 29% (7), BB 7% (3), D 0% (2), EPL 7% (2), FCU 7% (1)
Kim et al. (2019) [29]	13	85% (13)	69% (13)	31% (5)	EIP 46% (9), BB 0% (10), D 0% (8), ED 15% (5), FCU 38% (12), FPL 23% (4), PSP 0% (13)
Tsao et al. (2014) [14]	32	~50% (NA)	~33% (NA)	~33% (NA)	OP ~50% (NA), PSP 0% (28), Triceps 0% (31)
Seror et al. (2004) [32]	16	25% (16)	12% (NA)	NA	NA

Abbreviations: APB: abductor pollicis brevis; FDI: first dorsal interosseous; ADM: abductor digiti minimi; EIP: extensor indicis proprius; ED: extensor digitorum communis; FCR: flexor carpi radialis; BB: biceps brachii; D: deltoid; EPL: extensor pollicis longus; FCU: flexor carpi ulnaris; FPL: flexor pollicis longus; PSP: paraspinalis; OP: opponens pollicis; NA: data not available.

## Data Availability

Not applicable.
